# Effectiveness of a parenting programme in a public health setting: a randomised controlled trial of the positive parenting programme (Triple P) level 3 versus care as usual provided by the preventive child healthcare (PCH)

**DOI:** 10.1186/1471-2458-10-131

**Published:** 2010-03-15

**Authors:** Willem Spijkers, Daniëlle EMC Jansen, Gea de Meer, Sijmen A Reijneveld

**Affiliations:** 1 Department of Health Sciences, University Medical Center Groningen, University of Groningen, Antonius Deusinglaan 1, 9713 AV Groningen, the Netherlands; 2 Municipal Health Service Groningen, Hanzeplein 120, 9713 GW Groningen, the Netherlands; 3 Department of Sociology, Faculty of Behavioral and Social Sciences, University of Groningen, Grote Rozenstraat 31, 9712 TG Groningen, the Netherlands; 4 Municipal Health Service Fryslân, Harlingertrekweg 58, 8913 HR Leeuwarden, the Netherlands

## Abstract

**Background:**

Considering the high burden of disease of psychosocial problems in children and adolescents, early intervention regarding problem behaviour of young children is very important. The Preventive Child Healthcare (PCH) offers a good setting to detect such problem behaviour and to provide parenting support to the parents concerned. This paper aims to describe the design of an effectiveness study of a parenting programme for parents of children with mild psychosocial problems after an initial, evidence based screening in routine PCH.

**Methods/Design:**

The effects of the intervention will be studied in a randomised controlled trial. Prior to a routine PCH health examination, parents complete a screening questionnaire on psychosocial problems. Parents of children with increased but still subclinical levels of psychosocial problems will be assigned at random to the experimental group (Triple P, level 3) or to the control group (care as usual). Outcome measures, such as problem behaviour in the child and parenting behaviour, will be assessed before, directly after and 6 and 12 months after the intervention.

**Discussion:**

Parenting support may be an effective intervention to reduce psychosocial problems in children but evidence-based parenting programmes that fit the needs of the PCH are not available as yet. Although the Triple P programme seems promising and suitable for a universal population approach, evidence on its effectiveness in routine PCH still lacks.

**Trial registration:**

NTR1338

## Background

Psychosocial problems (e.g. aggressive behaviour, fear, anxiety) frequently occur in children and may lead to serious restrictions in daily functioning currently and in later life, and are the major cause of long-term work disability in young adults [1-3]. Several population-based studies in the Netherlands show that about 20% of all children struggle with psychosocial problems [4,5]. A study in primary and secondary education showed that 13% of all pupils had internalising problems, 11% had externalising problems and 3% had other behavioural problems [6].

Parenting style and child well-being are closely connected which makes parenting support a suitable way to decrease psychosocial problems in young children. Parents can help to prevent these problems in children by teaching them social skills [7]. Therefore methods for early treatment of psychosocial problems in children by enhancing parenting skills become increasingly available [8], but no evidence-based programmes for parenting support are available that suit the child healthcare. Only a minority of the children (13%) with psychosocial problems is under treatment by youth care or youth mental care [4,5,9] whereas early detection and treatment of psychosocial problems in children can improve their prognosis substantially ('the earlier, the better') [4,10,11].

In the Netherlands, Preventive Child Healthcare (PCH) offers an ideal opportunity for the early detection of psychosocial problems among preschool children, comparable to community pediatrics in the USA. In this system, child health professionals (further: CHP), i.e. doctors and nurses, working in preventive child healthcare offer routine well-child care clinics, including the early detection and treatment of psychosocial problems to the entire Dutch population [12,13]. Access is free of charge. This offers an ideal setting to provide parenting support following an evidence-based method of early detection of psychosocial problems in children. For this aim, there is a need for standardised parenting support interventions that are short and suit the competences of CHPs.

Triple P level 3, the so-called Primary Care Triple P, is such a short intervention but evidence for it after an initial screening on psychosocial problems in children in a preventive child healthcare setting still lacks. The intervention consists of practical advice and coaching on managing a specific behavioural problem during four short, individual consultations (20-30 minutes) with parents and their child by a trained child healthcare nurse. It is part of a multilevel system of early intervention for parents of children who have or are at risk of developing behavioural or emotional problems which aims at preventing and decreasing psychosocial problems in children by providing parenting support [14,15]. Research showed that Triple P, including level 3, seems promising when compared with a wait-list control group that receives no help [16]. A quasi-experimental study on the effects of Triple P level 3 in the Netherlands [17] showed significant decreases in the emotional and behavioural problems of children just as effects on parental satisfaction, parental efficacy and overall parental sense of competence. However, a randomised controlled trial investigating the effects of parenting support after an evidence-based, initial screening on psychosocial problems in children has never been done before and long-term follow-up data is currently not available.

### Objective

This paper aims to describe the design of an effectiveness study of a parenting programme for parents of children with mild problem behaviour after an initial screening in routine PCH.

## Methods/Design

### Design

The study is designed as a randomised controlled trial (RCT) with follow-up assessments directly after completion of the intervention, after six months and after twelve months. Eligible families will be randomly assigned to either the Triple P intervention or the care as usual (control) condition. The Medical Ethics Committee of the University Medical Center of Groningen approved the study design, protocols, procedures and informed consent. Participation is voluntary and all participants sign an informed consent form. In order to describe the design of this study, the CONSORT statement is followed [18], a checklist that intends to improve the quality of the reporting of randomised controlled trials.

### Participants

Eligible participants for the trial will be selected from a community sample of parents of 9-11 year old primary school children in the four northern provinces of the Netherlands, who will be examined during their routine PCH screening,. In the Netherlands, the PCH examines almost all children (>90%) at regular times. The four Northern provinces cover approximately 13% of all Dutch children of this age [19].

### Recruitment of study population

**Figure 1:**
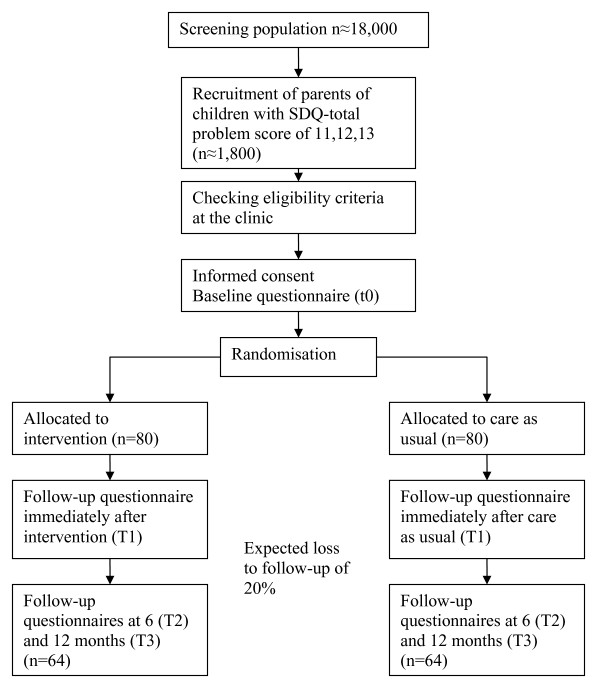
**Flowchart of participants**.

Prior to the contact with the PCH, all parents will be asked to complete a screening questionnaire on psychosocial problems in children. As part of the study, we added a baseline questionnaire about parenting behaviour. During routine examination by the PCH, the SDQ (Strengths and Difficulties Questionnaire) [20] total problems score of all children will be computed. Eligible participants, i.e. parents of children with a subclinical SDQ total problems score of 11-13, will be identified and invited by the CHPs to participate in the trial. After informed consent, they will be randomly assigned to the intervention or care as usual group (Figure 1). Families included in the trial will be asked to nominate a primary participating partner who will attend the intervention and complete the accompanying questionnaires. However, both parents are welcome to attend the counseling sessions, which will be carried out at the clinic or at home.

### Study inclusion and exclusion criteria

The inclusion criteria are: 1) Age 9 to 11 years, 2) An SDQ total problems score in the subclinical range, i.e. 11-13 [21], 3) Parents must acknowledge mild problem behaviour in their child, 4) Parents are willing to work on their child's psychosocial problems.

The exclusion criteria are: 1) A diagnosis of developmental delay, developmental disorder (e.g. autism), conduct disorder or ADHD in the child, 2) Currently receiving treatment for behavioural problems, 3) A chronic disease for which three or more medical consultations in the past two months have been made, 4) Parental divorce, death or severe disease of someone to whom the child feels attached to (e.g. (grand)parent, sibling, friend, nanny) in the past two months, 5) Parents being in therapy for psychological or relationship problems, 6) Parents being unable to read or speak Dutch, 7) Severe and/or general behavioural or emotional problems beyond the scope of the Triple P level 3 intervention, 8) Suspected parental dysfunction, such as child maltreatment, psychiatric disease, alcohol or drug abuse.

### Randomisation procedure

At the clinic, a research assistant ensures that participants enrolled by CHPs are eligible or have to be excluded from the trial. Randomisation, based on a computer-generated randomisation programme, occurs at the level of individual children in sequence of entrance. To prevent unequal randomisation, participants are pre-stratified and randomised by centre using block randomisation (blocks of six).

### Sample size

The SDQ will be the primary outcome measure for the power calculation. For an average 3-point decrease in SDQ total problems score, i.e. a change from the subclinical to the normal range, SD = 6 at alpha = 0.05 (two-sided) and beta = 0.20, 64 pairs of children need to be included with an initial SDQ total problems score between 11 and 13. As a loss to follow-up rate of 20% is expected, an initial study population of 80 pairs of children is needed in each treatment group (total: 160). This suffices to show an effect size of 0.5 of the Triple P programme regarding parent-reported child psychosocial problems and parenting problems.

### Blinding

Participants do not know which research group they will be assigned to. Nurses carrying out the treatment cannot be blinded for the allocated treatment. Since all follow-up questionnaires will be sent by mail, no direct influence by the researchers or the CHPs is likely to occur. After randomisation, all participants receive a research code unknown by the researcher. Therefore, analysis of the data by the researcher will be blind.

### Intervention

The intervention to be evaluated is Triple P level 3. Triple P is a multilevel system of family intervention which provides five levels of intervention of increasing strength [22]. Triple P intervention at level 3 (Primary Care Triple P) is a brief, narrow-focus parent programme that is aimed at parents with specific concerns about their child's behaviour or development [23]. It combines advice, rehearsal and self-evaluation to teach parents to manage a discrete child problem behaviour during four individual consultations of 20-30 minutes with the parents and their child (Table 1) [24].

**Table 1:** Overview of Triple P level 3 session content

Session	Contents	Duration
1 Assessment of the presenting problem	Intake interview	15 - 30 minutes
		
	Options for intervention	
		
	Keeping track of the children's behaviour	

2 Developing a parenting plan	Feedback of assessment results	15 - 30 minutes
		
	Causes of child behaviour problems	
		
	Goals for change	
		
	Parenting plan (with active skills training)	

3 Review of implementation	Update on progress	15 - 30 minutes
		
	Refining parenting plan (with active skills training)	
		
	Identifying and overcoming obstacles	
		
	Other issues	

4 Follow-up	Update on progress	15 - 30 minutes
		
	Maintaining progress made	
		
	Other issues	

To ensure the quality of delivery, intervention nurses completed a two-day training course delivered by the Netherlands Youth Institute and the developers of the programme and accreditation in levels 2 and 3 of the Triple P method by an accredited practitioner prior to the start of the project. The professionals conducting the interventions are all nurses employed in the PCH daily practice. Professional adherence to the Triple P method is warranted through several supervision sessions with an accredited practitioner.

### Control condition

Families in the control condition will receive the usual care initiated by a CHP (i.e. a community nurse). Protocols on psychosocial problems of the PCH-organisations prescribe, in case of a deviant SDQ total problem score, that the CHPs verify the gravity of the situation with the parents and, sometimes, with the schoolteacher. If the parents acknowledge the behavioural problems in their child and experience parenting problems, the CHP will try to clarify the problem and provide parenting support (e.g. strategies, tips, tricks). Usually CHPs have three extra contacts at their disposal to provide parenting support. If the problem exceeds the expertise of the CHPs, the parents and child will be referred to a specialist (e.g. psychologist, psychiatrist, child welfare). All CHPs have at least a bachelor's degree in nursing, which means that they have completed four years of education. Most of them also have received a two year specialisation training in community nursing.

### Outcome measures

The primary outcome of the study is problem behaviour of the child after intervention, measured by the (1) Strengths and Difficulties Questionnaire (SDQ) [20], 30 items divided into 5 subscales on prosocial behaviour, hyperactivity, emotional symptoms, conduct problems and peer problems and (2) the Eyberg Child Behavior Inventory (ECBI) [25], 36 items on parental perception of disruptive behaviour including intensity and problem score.

The secondary outcome of the study is parenting behaviour since the intervention aims at parenting as mediator. Parental competence and parenting style will be measured by the (1) Problem Setting and Behaviour Checklist (PSBC) [26], a 28-item rating scale that assesses how confident parents are in dealing with child behaviour problems in various settings and (2) the Parenting Scale (PS) [27], 30 items on parenting behaviour (permissiveness, overreactivity, verbosity). Parenting stress will be measured by (1) the Dutch Parental Stress Index (PSI) [28], 11 items on parenting stress, and (2) the 21-item Depression, Anxiety and Stress Scale (DASS) [29].

At baseline family characteristics will be assessed, i.e.: family situation, age of the parents, parental education, employment and financial situation of the parents, and the ethnicity of the parents and the child.

### Data collection procedure

Data will be obtained by questionnaires. Participants will be asked to return each of the questionnaires within one week. To minimise loss to follow-up, the parents will be called by phone if the questionnaire has not been returned within one week. The outcome assessments will take place, regard for equal time intervals, directly after treatment (T1), six months (T2) and twelve months (T3) after treatment. Parents who complete all questionnaires will receive a gift voucher.

### Analysis

Statistical analysis will be performed according to the 'intention-to-treat' principle. With multiple measurements over time, data on subjects lost to follow-up will be handled by imputation techniques. Change in child behaviour and parenting behaviour will be expressed as standardised effect sizes. To analyse the development of the outcome measures in time, a longitudinal data analysis technique, i.e. random coefficient analysis, will be applied. Baseline characteristics of the parents in the two research groups will be compared using Chi-squared tests for categorical variables, Wilcoxon's test for ordinal variables, and t-tests for continuous variables. Reporting will follow the CONSORT guidelines.

### Time frame of the study

The preparatory period will take six months. In this period, nurses will be trained to carry out the intervention (Triple P, level 3). Furthermore, CHPs will be trained in recruiting potential participants in the trial. The inclusion phase will last two years and the follow-up phase will be twelve months. Analysing data and reporting the findings will last for six months. Therefore, the total duration of the study will be four years.

## Discussion

This paper presents the design of a randomised controlled trial to investigate the effectiveness of a parenting programme (Triple P level 3) for parents of children with psychosocial problems after an initial, evidence based screening in routine PCH. Research on the effectiveness of interventions in routine PCH is very scarce [8] and no previous trial has been accomodated in a further evidence-based procedure.

Studying the effects of this intervention is important as it aims (1) to reduce the burden of disease of psychosocial problems in children [4,5] and (2) to contribute to the use of evidence-based care for children with psychosocial problems. Furthermore, introducing an evidence-based programme may lead to a more recognisable and consistent approach to parenting support by the PCH.

This short intervention can be easily embedded in the regular procedure. If CHPs suspect parenting problems or behavioural problems in children due to incompetent parenting, they can offer help to parents in three extra contacts as is customary. If Triple P level 3 proves to be effective, this will be conducive to further implementation. CHPs as well as parents of children with mild problem behaviour may benefit from a structured approach to working on and solving problem behaviour in young children. If proven effective, level 3 of the Triple P programme in combination with an available evidence-based detection of psychosocial problems in children may lead to a comprehensive evidence-based method to reduce the burden of disease due to psychosocial problems. Governmental organisations and policymakers may use the results of this study to develop future policy concerning parenting support provided by community healthcare workers.

### Strengths and limitations

A randomisation procedure is applied to reduce the risk of selection and allocation bias. Since participants do not know each other, mutual influencing is considered unlikely. A broad array of outcome measurements gains insight into the treatment effect in many areas of parenting and child behaviour. Furthermore, this study is original in evaluating the effectiveness of an intervention on parenting support in a preventive healthcare organisation (i.e. the Dutch PCH) delivered with regular staff from multiple centres. While the intervention will be carried out in the daily PCH practice, conclusions can be generalised without reservations which makes the external validity of the trial strong. The intention-to-treat analysis gives the trial high internal validity. Contrary to earlier studies, in this research the long-term effects will be assessed by measurements after twelve months after treatment.

There are also some limitations. This study does not provide an independent, professional evaluation of psychosocial problems in children apart from parental judgement. However, parental concerns are a good indicator of problems indentified by professional [30] and observational research is difficult to objectify. Furthermore, the effects of the intervention may depend on the CHPs affinity for parenting support and treatment adherence. Assessment of the effects of parenting support at the level of independent health care workers within this study would be to laborious.

The results of this study will become available in 2012.

## Competing interests

The authors declare that they have no competing interests.

## Authors' contributions

SAR, GdM and WS had the original idea for the project, wrote the study proposal, and obtained the funding of the study. WS and DEMCJ wrote the study protocol which was discussed by all authors leading to the final design. WS wrote the final manuscript, which was discussed, edited and revised by all authors. All authors read and approved the final manuscript.

## Pre-publication history

The pre-publication history for this paper can be accessed here:

http://www.biomedcentral.com/1471-2458/10/131/prepub
